# Genetic Diversity and Ethnic Tapestry of Kazakhstan as Inferred from HLA Polymorphism and Population Dynamics: A Comprehensive Review

**DOI:** 10.3390/genes16030342

**Published:** 2025-03-15

**Authors:** Aida Turganbekova, Saniya Abdrakhmanova, Zhaksylyk Masalimov, Wassim Y. Almawi

**Affiliations:** 1 Scientific and Production Center for Transfusiology, Astana 010000, Kazakhstan; dr.aida@mail.ru (A.T.); a.saniya@mail.ru (S.A.); 2 Faculty of Natural Sciences, L.N. Gumilyov Eurasian National University, Astana 010000, Kazakhstan; massalimov@gmail.com; 3 Faculty of Sciences, El-Manar University, El-Manar University Campus at El-Manar, Tunis 2092, Tunisia

**Keywords:** genetic diversity, HLA, immunogenetics, Kazakhstan, linkage disequilibrium, polymorphisms

## Abstract

**Background:** The human leukocyte antigen (HLA) system represents the most polymorphic segment within human DNA sequences and constitutes a core component of immune defense responses and in understanding population genetics. This research investigates the distribution of HLA class I and II polymorphisms across different ethnic groups in Kazakhstan, offering valuable insights into the genetic diversity and demographic evolution within this region. **Methods:** We performed an in-depth examination of HLA class I and II polymorphisms across diverse ethnic communities living in Kazakhstan, including Kazakhs, Russians, Uzbeks, Ukrainians, Germans, Tatars, and Koreans. Utilizing data from high-resolution HLA typing studies allowed us to assess allele frequencies alongside haplotype distributions while analyzing genetic interrelations between these populations. Additionally, we performed comparative assessments with global HLA databases to determine the genetic affiliations between these groups and their relationships with neighboring and more distant populations. **Results:** Our study revealed over 200 HLA alleles within the analyzed populations, and significant variations were observed in their allele and haplotype frequencies. Notably, the Kazakh group exhibited strong genetic ties to Asian and Siberian demographics; conversely, other ethnicities showed associations reflective of their historical roots. Notable alleles included HLA-A*02:01, B*07:02, C*07:02, DRB1*07:01, and DQB1*03:01, commonly observed across various groups. Linkage disequilibrium analysis revealed the presence of population-specific haplotypes, highlighting distinct genetic structures within these communities. **Conclusions**: The findings highlight the significant genetic diversity in Kazakhstan, influenced by its geographical location at the crossroads of Europe and Asia. These results are pertinent to immunogenetics, transplantation medicine, and personalized healthcare within Kazakhstan and adjacent regions. Future research should expand the sample size and explore disease associations to enhance our comprehension of HLA genetics across Central Asia.

## 1. Introduction

The human leukocyte antigen (HLA) system represents the most diverse genetic framework known to date [1,2]. As of September 2024, 39,627 alleles linked to HLA genes have been identified. These comprise 28,409 from class I and 12,594 from class II, of which the B locus is the most variable with 10,346 alleles (http://hla.alleles.org). This region resides on the short arm of chromosome 6 at position p21.3 and encompasses over 220 distinct genes that serve various functions [2,3], and consists of classes I (HLA-A, B, and C) and II (HLA-DR, DQ, and DP) [2,4,5,6].

The organization and functional diversity within the HLA system are further shaped by linkage disequilibrium, which dictates the non-random associations among alleles across the same gene class or nearby loci [2,7]. Also referred to as haplotype transfer, it affects more than a hundred neighboring genes separated by approximately 3.6 Mb (https://hla.alleles.org). Functionally, in addition to their role in controlling immune responses and influencing the development of autoimmune disorders [4,8], HLA alleles play a crucial role in population studies due to their unique distribution patterns across different ethnic groups [9], and thus serve an anthropological role [3,6].

## 2. Molecular Structure of HLA System

While HLA molecules demonstrate codominant expression, their distribution is not uniform across various tissues [9]. Class I HLA molecules (A, B, C) comprise a 45 kDa α-chain that non-covalently associates with the smaller 12 kDa β2-microglobulin and are predominantly located in nearly all nucleated cells, platelets, and immature erythrocytes; they are essential for presenting antigens to cytotoxic CD8+ T cells. Conversely, class II HLA molecules (DP, DQ, DR) comprise a 33 kDa α-chain non-covalently paired with a 28 kDa β-chain throughout their extracellular regions [10]. Primarily found on specialized antigen-presenting cells, including monocytes/macrophages, endothelial cells, B cells, and dendritic cells, class II molecules can be expressed or upregulated under inflammatory conditions by various cell types like T cells and endothelial components. Their primary function is to present external antigens to helper CD4+ T cells [11]. The immune-modulating functions of both classes of HLAs are executed during antigen presentation through intricate interactions between processed peptides recognized by T-cell receptors (TCRs), which arise from selective coreceptor binding. While CD4 interacts with class II β-chains, CD8 specifically pairs with class I α-chains [10].

## 3. HLA System Genetics, Inheritance, and Nomenclature

HLA genes are closely linked within the highly polymorphic MHC on chromosome 6p21.3. The class I region consists of HLA-A, HLA-B, and HLA-C, while the class II region (HLA-D) encodes the α- and β-chains of HLA-DR, HLA-DP, and HLA-DQ. Because of this tight genetic linkage, HLA genes are often inherited as a single haplotype with rare genetic crossovers [12]. This linkage disequilibrium (LD) allows for predictable allele associations and facilitates accurate HLA genotype estimation [13].

HLA genes demonstrate extensive polymorphism, resulting from diverse factors such as mutations, recombination, and gene conversion. This is especially evident in the antigen-binding domains of class I (α-1, α-2) and class II (α-1, β-1) [12]. Such variability is crucial in immune regulation, dictates susceptibility to autoimmune diseases, and influences tissue compatibility and drug hypersensitivity [4,5,6]. Over 20,000 class I and 10,000 class II variants define unique peptide-binding motifs, enabling diverse antigen recognition [14]. To manage this complexity, the WHO Nomenclature Committee standardizes HLA gene naming. Molecular typing, indicated by an asterisk (*), categorizes alleles into hierarchical fields (e.g., HLA-A*02:01). The first field denotes the antigen group, and the second specifies unique proteins. In contrast, subsequent fields capture silent mutations and non-coding polymorphisms affecting gene expression [15].

## 4. HLA Typing Methods

HLA typing is utilized in various clinical fields, such as transplantation, transfusion medicine, and immunogenetics [3,5,6]. Historically, the determination of HLAs relied on sera with known antibody specificities [16,17]. The diversity of HLA genes has necessitated a transition to molecular typing techniques with varying levels of resolution [18]. Of these, “low resolution” (e.g., HLA-A*01) is considered adequate for areas where high accuracy may not be critical. On the other hand, “high-resolution” typing (e.g., HLA-A*01:02) enables allele identification through analysis of exon region sequences within the HLA molecule, which are reflected in the first two fields in the nomenclature format [18,19]. Notably absent from this level are synonymous mutations and variations occurring in non-coding regions [18].

Currently, multiple technologies are available for HLA typing. These include oligonucleotide probe hybridization (SSO), sequence-specific primer polymerase chain reaction (SSP), allele-specific PCR (AS-PCR), real-time PCR, DNA sequence-based typing (SBT)**,** next-generation sequencing (NGS), and nanopore sequencing [17,18]. The selection of a particular method depends on the specifics of the laboratory. SSP typing is particularly advantageous for low-volume applications and single-typing scenarios in contexts like organ transplantation from living-related individuals or postmortem donors. On the other hand, SSO is preferred for mass typing efforts to create databases such as patient waiting lists for organ donations or bone marrow donor registries [18,19]. However, it should be noted that these techniques typically offer lower resolution. In contrast, NGS and nanopore sequencing provide high-resolution data suitable for larger cohorts; nevertheless, SBT remains the gold standard for high-resolution typing [20,21,22].

## 5. Comprehensive HLA Database Resources

The IPD-IMGT/HLA database has been a repository of polymorphisms of immune system genes since its inception in 1998 when the database on 964 allelic variants was first released [23]. The IPD-IMGT/HLA database contains an extensive collection of allelic variants and remains a vital resource for HLA investigations (https://www.ebi.ac.uk/ipd/imgt/hla/, accessed on 12 January 2025). Beyond the sequences, the database provides detailed information regarding the origin of these sequences, along with validation data [24]. Contributors submit their sequences directly to IPD-IMGT/HLA for curation and official nomenclature before publication, thereby mitigating the issues related to renaming published sequences and alleviating confusion stemming from multiple designations assigned to identical sequences [23,24].

In collaboration with organizations such as the Imperial Cancer Research Fund (now known as Cancer Research UK) and the European Bioinformatics Institute, Oracle^®^ databases have been established that facilitate advanced queries on HLA sequences through an online graphical user interface [14,15,24]. The Allele Frequency Net Database (AFND) is another critical HLA database. This centralized registry analyzes variations in the frequencies of HLA genes in different populations worldwide [25].

The World Marrow Donor Association (WMDA) is one of the most extensive repositories for HLA phenotypes, encompassing over 41 million registered donors and more than 750,000 cord blood units from 57 countries (https://statistics.wmda.info/). The database was created to find suitable donors of hematopoietic stem cells for transplantation [26]. Kazakhstan’s National Registry has also been included in the WMDA since 2021 and has contributed about 4000 HLA phenotypes of voluntary HSC donors [26].

Another extensive system, the Immuno Polymorphism Database (IPD), describes the diversity of immune system gene polymorphisms and contains more than 30,000 HLA alleles in the IPD-IMGT/HLA section (http://www.ebi.ac.uk/ipd/, accessed on 12 January 2025). The IPD consists of specialized databases that examine polymorphisms in immune genes and works with nomenclature committees that provide and curate sections [27]. The IPD includes six key components:

- IPD-IMGT/HLA: Originally part of the international ImMunoGeneTics (IMGT) project, it houses HLA sequences, including the official entries designated by the WHO Nomenclature Committee for Factors of the HLA System.
- IPD-KIR: Allelic sequences of immunoglobulin-like killer cell receptors.
- IPD-MHC: Sequences of major histocompatibility complexes of different species.
- IPD-NHKIR: A centralised repository for non-human KIR (NHKIR) sequences.
- IPD-HPA: A centralised repository for the allo-compatible human platelet antigens (HPAs).
- IPD-ESTDAB (European Tumor Cell Database): Provides for online searches on HLA-typed, immunologically characterized tumor cells.

Data generated by these resources are available on their websites and via an FTP catalogue [27,28].

## 6. Frequent HLA Alleles in the Population of Kazakhstan

**Figure 1:**
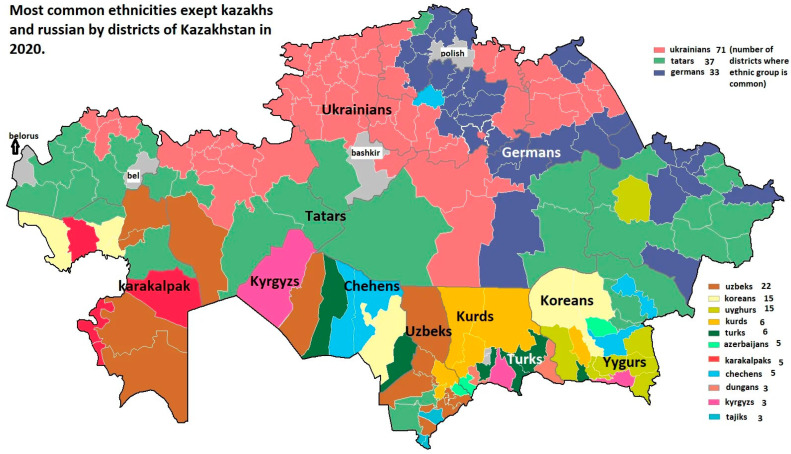
National clustering of common ethnicities, except Kazakhs and Russians, by district of Kazakhstan in 2020.

Kazakhstan is a transcontinental country that lies across Central Asia and Eastern Europe and ranks the ninth-largest country worldwide, with an area of 2,724,900 km^2^. The present-day population of Kazakhstan (est. 20 million) is characterized by multinational/multiethnic origin, primarily of Turkic origin [29]. According to 2023 data, ethnic Kazakhs constitute 70.6% of the populace, followed by Russians (15.1%), Uzbeks (3.2%), Ukrainians (1.9%), Uighurs (1.5%), Germans and Tatars (each 1.1%), Azerbaijanis (0.7%), Koreans (0.6%), and other nationalities [30,31] (Figure 1).

**Figure 2:**
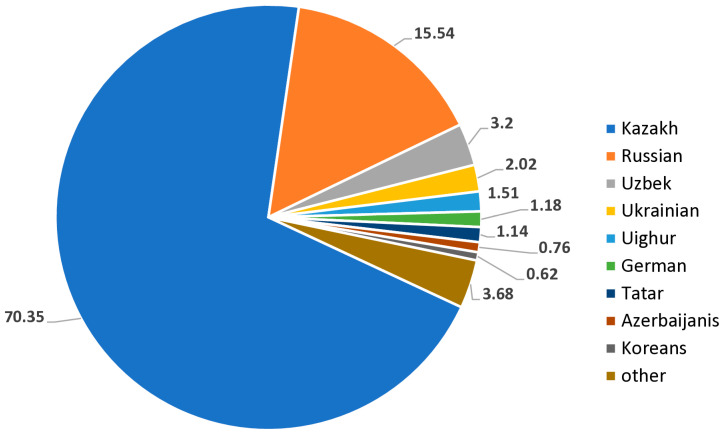
The distribution of the ethnic groups varies across different regions of Kazakhstan.

The distribution of these ethnic groups varies across different regions of Kazakhstan (Figure 2):

- The Turkestan region is home to the most ethnic Kazakhs (1.5 million) and Uzbeks (378,000).
- Most Russians (428,000) are found in the Almaty region (South Kazakhstan).
- The Kostanay region is home to most Kazakhstani Ukrainians (86,000).
- Nearly all Uighurs (297,000) live in the Almaty region [32].

These unique demographics highlight the historical background and cultural diversity unique to the Kazakhstani population [29].

## 7. HLA Profile of Russian Population of Kazakhstan

Our recent study involving 947 individuals of Russian descent residing in Kazakhstan revealed 216 unique HLA class I and II alleles. The most common class I alleles were A*02:01 (26.5%), B*07:02 (11.1%), C*04:01 (13.5%), and C*06:02 (12.1%). In contrast, DRB1*07:01 (13.8%), DRB1*15:01 (12.2%), and DQB1*03:01 (19.7%) were the predominant class II alleles [31]. Notably, significant linkage disequilibrium was detected across all identified HLA loci. The most prevalent two-locus haplotypes comprised DRB1*15:01~DQB1*06:02 (10.5%), B*07:02~C*07:02 (10.0%), B*07:02~DRB1*15:01 (6.3%), and A*01:01~B*08:01 (4.5%). Furthermore, the most frequent five-locus haplotypes identified were A*01:01~C*07:01~B*08:01~DRB1*03:01~DQB1*02:01 (3.3%) and A*03:01~C*07:02~B*07:02~B*07:02~DRB1*15:01~DQB1*06:02 (2.3%) [31].

Based on their unique HLA profile and subsequent neighborhood joining (NJ) trees and standard genetic distance (SGD) analysis, we demonstrated a close relationship between Kazakh Russians and various Western Russian groups, including Northwest Slavs (Vologda, Chelyabinsk, Moscow), Eastern Europeans (Belarus-Brest, Ukraine, Poland), and Scandinavians (Swedes, Finns) [33]. In contrast, notable differences were seen with Eastern Russian populations, in particular Tuvinians and Siberians hailing from Chukotka, Kamchatka, and Ulcha, as well as groups from the Eastern Mediterranean region, including Levantines, Turks, Northern Macedonians, and Albanians, and East Asian populations, such as Koreans, Japanese, Taiwanese, and Mongolians [33,34]. These results corroborate historical data, suggesting that migrants from Russia who settled in Central Asia mainly came from European Russia during waves of migration throughout the 18th and 19th centuries [31,34].

## 8. HLA Profile of Uzbek Population of Kazakhstan

The Uzbeks constitute the second-largest ethnic group within Kazakhstan. Examination of their HLA profile identified 119 HLA alleles, with A*02:01 (17.86%), B*07:02 (8.33%), and C*04:01 (15.00%) being the most frequent class I alleles, while DRB1*07:01 (13.09%) and DQB1*03:01 (20%) were the most prevalent class II alleles [30]. The most frequent five-locus haplotype identified in the Uzbek population was A*03:01~B*07:02~C*07:02~DRB1*15:01~DQB1*06:02 (6.00%), followed by A*24:02~B*38:01~C*12:03~DRB1*14:01~DQB1*05:03 (3.75%) [30]. In addition, two-locus haplotype analysis identified A*02:01~B*38:01 (5.0%), B*13:02~DRB1*07:01 (6.3%), B*49:01~C*07:01 (7.5%), DRB1*15:01~DQB1*06:02 (8.8%), DRB1*14:01~DQB1*05:03, and DRB1*07:01~DQB1*02:01 (8.8%) as the most common haplotypes. This demonstrates that Uzbeks living in Kazakhstan have comparable HLA profiles to Siberians and Eastern Russians, suggesting genetic relatedness to Southeastern Russians, Eastern Europeans, and Asian populations [35]. Despite Uzbekistan’s location between Europe and Asia, there appear to be notable genetic similarities between Uzbeks and native Kazakhs [30,35].

## 9. HLA Profile of Ukrainian Population of Kazakhstan

Ukrainians rank as the fourth-largest ethnic group in Kazakhstan, following native Kazakhs, Russians, and Uzbeks. An examination of their HLA profile identified a total of 128 HLA alleles, of which A*02:01 (28.64%), B*07:02 (9.71%), B*13:02 (9.71%), and C*06:02 (15.05%) were the most prevalent class I alleles, while DRB1*07:01 (15.20%) and DQB1*03:01 (24.76%) were the most frequent class II alleles [36]. The most prevalent two-locus haplotypes were A*02:01~B*13:02 (6.66%), B*13:02~DRB1*07:01 (6.57%), B*13:02~C*06:02 (10.10%), and DRB1*01:01~DQB1*05:01 (12.12%). In addition, the haplotype *A*02:01*~*B*13:02~C*06:02*~*DRB107:01~DQB1*02:01 emerged as the most prevalent five-locus combination, accounting for 4.04% of cases [36]. Comparative analyses indicate that Ukrainians residing in Kazakhstan share a closer genetic affiliation with Eastern Europeans and European Russians while exhibiting significant divergence from Asian populations [36,37]. This supports the historical perspective that Kazakhstani Ukrainians trace their roots back to Ukraine, following large-scale migrations to Central Asia during the eighteenth and twentieth centuries [36,37].

## 10. HLA Profile of German Population of Kazakhstan

The German population in Kazakhstan is primarily located in the northeastern regions. The history of the Kazakhstani Germans traces back to the 18th century when Russian settlers moved to the Volga region but relocated to Kazakhstan in the early 20th century. Analysis of the HLA profile among Kazakhstani Germans identified 107 HLA alleles comprising 16 HLA-A, 33 HLA-B, 21 HLA-C, 21 HLA-DRB1, and 16 HLA-DQB1 alleles. Of these, the most common class I alleles were A*02:01 (25.49%), B*07:02 (9.80%), B*08:01 (9.80%), C*07:02 (13.46%), and C*06:02 (12.5%), while DRB1*07:01 (21.57%) and DQB1*03:01 (25.00%) were the most prevalent class II alleles [9,20]. Comparable frequencies of these alleles were also reported for Germans, European Russians, and Eastern Europeans [9,37]. In addition, A*03:01~B*07:02 (6%), B*07:02~DRB1*15:01 (8%), B*07:02~C*07:02 (10%), DRB1*15:01~DQB1*06:02 (12%), and DRB1*07:01~DQB1*02:01 (10%) were the most common two-locus haplotypes, while A*03:01~B*07:02~C*07:02~DRB1*15:01~DQB1*06:02 (6%) and A*01:01~B*08:01~C*07:01~DRB1*03:01~DQB1*02:01 (5%) were the most frequent five-locus haplotypes among Kazakhstani Germans [30]. These haplotypes are also prevalent in European Russians [37].

Genetic SGD analysis showed that Kazakhstani Germans share the closest genetic ties with South Ural Tatars, Volozhans, other Tatar groups, Poles, and ethnic Germans themselves [30,37]. Kazakhstani Germans display genetic similarities with Europeans from Russia and Eastern Europe’s populations. Furthermore, genetic relatedness was reported for Aleuts (Bering Island), Buryats, Belarusian individuals from Brest, and Tuvinians compared to Kazakhstani Germans and Kazakhstani Uzbeks [30]. Interestingly, while Kazakhstani Germans exhibited genetic similarities with European Russians, Germans, and Eastern Europeans, the Uzbek minority was more closely related to Southeastern Russians, Eastern Europeans, and certain Asian populations [9,30,37].

## 11. HLA Profile of Tatar Population of Kazakhstan

The Tatars constitute a distinct ethnic minority within Kazakhstan, accounting for 1.3% of the total population [38]. Our recent study on 103 Kazakhstani Tatars identified 132 HLA alleles, of which A*02:01 (20.1%), B*07:02 (12.1%), and C*07:02 (12.7%) were the most frequent class I alleles, while DRB1*07:01 (18.1%) and DQB1*02:01 (19.6%) were the most prevalent class II alleles (Hajjej et al., 2024 [39]). B*07:02~C*07:02 (10.6%), B*07:02~DRB1*15:01 (06.1%), B*07:02~DQB1*06:02 (7.01%), and DRB1*15:01~DQB1*06:02 (11.6%) were the most frequent two-locus haplotypes, and extended five-locus analysis identified A*01:01~B*08:01~C*07:01~DRB1*03:01~DQB1*02:01 (4.0%), and A*02:01~B*07:02~C*07:02~DRB1*15:01~DQB1*06:02 (3.2%) as the most frequent haplotypes [39]. These haplotypes showed similar frequencies to those in our previous work on Russians residing in Kazakhstan [31] and other populations from Eastern Russia [37], with slight differences noted [25]. Historical evidence suggests a close genetic relatedness of Kazakhstani Tatars with European Russians, particularly Russian Tatars, which supports the notion that Kazakhstani Tatars may have descended from Russian Tatars located in the Volga region before their mass migrations to Central Asia during periods of marked population movement [9,39].

## 12. HLA Profile of Korean Population of Kazakhstan

The Korean population in Kazakhstan is recognized as the eighth-largest ethnic minority within the country. Analysis of HLA genotypes revealed 131 distinct HLA alleles, with the most common class I alleles being A*02:01 (23.08%), B*35:01 (8.24%), and C*01:02 (15.38%). In terms of class II alleles, *DRB1*08:03 (9.89%) and DQB1*03:01* (21.98%) were identified as predominant [40]. The haplotype assessment identified A*03:01∼B*07:02 (3.85%), B*08:01∼DRB1*03:01 (3.85%), *B*07:02∼C*07:02* (7.14%), and *DRB1*08:03∼DQB1*06::0(9)34% as the leading two-locus haplotypes among Koreans in this region. In addition, A*02:01∼B*18:01∼C*07:01∼DRB1*11:04∼DQB1*03:01 and A*33:03∼B*44:03∼C*14:03∼DRB1*13:02∼DQB1*06:04 (at 2.2% for each) were noted as the most frequent five-locus haplotypes [3]. SGD analysis demonstrated that Kazakhstani Koreans are genetically closely related to East Asian populations, such as Mongols (SGD, 0.044), Eastern Siberian Tuvinians (SGD, 0.081), and Siberian Buryats (SGD, 0.094) [9,30,37]. In contrast, they are genetically distant from populations originating from the Eastern Mediterranean region, such as Lebanese (SGD, 0.367), Greek (SGD, 0.377), and Saudis (SGD, 0.414), and most Siberians (SGD, 0.473–0.699) [40]. This relatedness is attributed mainly to the mass migration, known as Koryo Saram, to Kazakhstan in the 20th century [39].

## 13. Distribution of HLA Alleles Among Kazakh Population of Kazakhstan

The Kazakh ethnic group comprises more than 70% of the present-day population of Kazakhstan. However, information about the HLA profile of the native Kazakh population remains scarce. Here, we consolidate available data on the genetic makeup of the native Kazakh community and associated historical influences. Historically, the origin of the Kazakhs traces back to the 1st millennium AD, with formal recognition as a separate entity, as the Kazakh Khanate, established in the 15th century [41]. Kazakhstan is in Central Asia, at the crossroads between Europe and Asia, and is characterized by diverse languages, religions, and cultures [31,42].

The invasions of the Mongols significantly contributed to shaping anthropological characteristics and genetic diversity among the present-day Kazakh population, who are descendants of Turkic tribes and Turkic-Mongol tribes [42,43]. An earlier study reported on the HLA-DRB1, -DQA1, and -DQB1 profiles of 314 healthy individuals residing in Astana city as well as Tarbagatai district in East Kazakhstan, utilizing sequence-based typing (SBT) [35]. Neighbor-joining dendrogram analysis, based on HLA-DRB1 allele frequencies in Kazakh and other populations, demonstrated that native Kazakhs cluster closely with various Asian and Siberian groups but remain distinctly separate from European populations such as Scandinavians or those originating from Mediterranean regions [35]. Multidimensional scaling (MDS) analysis validated this. It established strong genetic connections between present-day Kazakhs and Chinese, such as Uighurs, and other ethnic groups like Mongols, Tojins, and Tuvinians, together with additional Siberian and Asian communities [35]. This underscores the deep-rooted genetic linkages that connect Kazakhstan to Central and Northern Asia while providing insights into the dynamics of ancestry and migration trends.

Genotyping of the HLA class II DRB1, DQA1, DQB1, and DPB1 genes was performed on 42 healthy individuals of both genders from the Kazakh population residing in Urumqi, located in Xinjiang Uygur Zizhiqiu, the northwestern region of China [44]. The most frequent genes identified were DRB1*0301 (13.1%) and DRB1*07 (10.7%), DQA1*0501 (29.8%) and *DQAP0301* (23.8%), DQB1*0201 (23.8%) and DQB1*0301 (21.4%), and DPB1*0401 (21.4%), DPB1*0501 (20.2%), DPB1*0402 (19.0%), and DPB1*0201 (16.7%). A dendrogram was constructed using the neighbor joining (NJ) method based on the allele frequencies of the DRB1, DQA1, DQB1, and DPB1 genes [44]. That study, which included 12 different populations besides the Kazakhs, such as Northern and Southern Han Chinese, Manchu, and Japanese, demonstrated that Kazakhs share closer genetic relatedness with Northwestern Han than other Asian ethnic groups [44].

A comprehensive high-resolution HLA genotype analysis was conducted on 5800 potential hematopoietic stem cell donors registered in the National Register of the Republic of Kazakhstan. The analysis examined HLA-A, HLA-B, HLA-C, HLA-DRB1, and HLA-DQB1 loci, demonstrating marked diversity in the genetic profile within the Kazakh population. Significant allelic diversity was revealed, and most were heterozygous. For class I loci, 101 allele groups were identified for the HLA-A locus, 155 for the HLA-B locus, and 73 for the HLA-C locus (Table 1). For class II loci, there were 94 allele groups for the HLA-DRB1 locus and 41 for the HLA-DQB1 locus (Table 1). The high frequency of heterozygosity suggests a genetically diverse population, which has implications for immune response, disease susceptibility, and donor compatibility.

The most frequent class I alleles were (A locus) A*02:01 (18.7%), A*24:02 (15.3), and A*01:01 (10.1%); (B locus) B*51:01 (8.9%), B*13:02 (6.2%), and B*07:02 (5.2%); and (C locus) C*06:02 (13.9%), C*04:01 (9.1%), and C*07:02 (8.8%). On the other hand, the most prevalent class II alleles were (DRB1 locus) DRB1*07:01 (13.1%), DRB1*03:01 (10.1%), and DRB1*15:01 (8.9%), and (DQB1 locus) DQB1*03:01 (23.5%), DQB1*02:01 (16.2%), and DQB1*05:01 (9.1%). A full list of the common (>1%) class I and class II alleles can be found in Supplementary Table S1.

While the HLA haplotype distribution revealed extensive heterogeneity among the native Kazakh population, distinct three-locus class I, two-locus class II, and five-locus extended class I–two-locus class II haplotypes were identified. The most frequent class I *A~C~B* haplotype was A*01:01~C*07:01~B*15:17 (1.6%), followed by A*02:01~C*07:02~B*08:01 and A*03:01~C*07:02~B*08:01 (each at 1.4%), A*02:01~C*06:02~B*14:02 (1.3%), and A*01:01~C*01:02~B*07:02 and A*03:00~C*04:01~B*35:02 (each at 1.1%) (Table 2). On the other hand, the most common *DRB1~DQB1* class II haplotype was DRB1*15:01~DQB1*06:02 (8.8%), followed by DRB1*03:01~DQB1*02:01 (7.2%), DRB1*11:01~DQB1*03:01 (5.2%), DRB1*13:01~DQB1*06:03 (5.0%), and DRB1*07:01~DQB102:01 (4.7%) (Table 2). For the extended (*A~C~B~DRB1~DQB1*) HLA haplotypes, the most prevalent two-locus haplotypes comprised A*01:01~C*07:01~B*06:01~DRB1*11:01~DQB1*03:01 and A*01:01~C*08:01~B*07:01~DRB1*03:01~DQB1*04:01 (each at 1.3%). Less common haplotypes included A*01:01~C*08:01~B*04:01~DRB1*07:01~DQB1*03:03 and A*02:01~C*13:02~B*06:02~DRB1*07:01~DQB1*02:02 (each at 0.5%) (Table 2).

Further genetic distance analysis is underway to examine how the Kazakh population compares to other ethnic groups, with likely applications in precision medicine and disease association investigations [45].

## 14. Overall Assessment and Significance

This in-depth examination of HLA polymorphisms among the diverse ethnicities of Kazakhstan provides significant insights into the genetic composition, variability, and ancestral connections that characterize its numerous ethnic groups. The identification of more than 200 distinct HLA alleles across various class I and class II loci, notably the common A*02:01, B*07:02, C*07:02, DRB1*07:01, and DQB1*03:01 alleles found at elevated frequencies among multiple ethnic communities, underscores the genetic diversity present within Kazakhstan’s populace. While (native) Kazakhs demonstrated a closer genetic affinity to Asian and Siberian populations, confirming the historical migration patterns and shared ancestry, the clustering of Kazakhstani Russians [31], Germans [30], and Ukrainians [36] with European cohorts is consistent with their Eastern European origins. Furthermore, Uzbeks revealed genetic relatedness to Eastern European and Asian populations, which reflects their intermediary geographic position between Europe and Asia [30].

Functionally, the high-resolution data presented in our studies offer key information for transplantation registries aimed at enhancing successful donor–recipient matching. This study aids in understanding the genetic predisposition to immune disorders linked to specific HLA alleles and haplotypes, including autoimmune and inflammatory disorders, as well as pharmacogenomics specifically pertinent to Kazakhstan’s population.

## 15. Concluding Remarks

The findings substantially affect population genetics, immunogenetics, and transplantation medicine. Further investigations into genetic distance analysis, functional HLA variations, and their impact on public health will enhance personalized medicine and donor-matching programs in Kazakhstan and beyond.

## Figures and Tables

**Table 1:** HLA diversity of Kazakh population.

Locus	Allele Number	% Heterozygous	% Homozygous	Common Alleles
HLA-A	101	86	14	A*02:01, A*24:02, A*01:01
HLA-B	155	92	8	B*07:02, B*51:01, B*13:02
HLA-C	76	89	11	C*06:02, C*07:02, C*04:01
HLA-DRB1	94	90	10	DRB1*07:01, DRB1*03:01, DRB1*15:01
HLA-DQB1	41	87	13	DQB1*03:01, DQB1*02:01, DQB1*06:03

**Table 2:** Most frequent HLA class I and class II haplotypes among native Kazakh population.

HLA Class	Haplotype	Frequency	Percent
*A~* *C~* *B*	A*01:01~ C*07:01~ B*15:17	30	1.6
	A*02:01~ C*07:02~ B*08:01	26	1.4
	A*03:01~ C*07:02~ B*08:01	26	1.4
	A*02:01~ C*06:02~ B*14:02	25	1.3
	A*01:01~ C*01:02~ B*07:02	20	1.1
	A*03:0~ C*04:01~ B*35:02	21	1.1
	A*26:01~ C*12:03~ B*39:01	18	1.0
*DRB1~* *DQB1*	DRB1*15:01~ DQB1*06:02	166	8.8
	DRB1*03:01~ DQB1*02:01	135	7.2
	DRB1*11:01~ DQB1*03:01	98	5.2
	DRB1*13:01~ DQB1*06:03	93	5
	DRB1*07:01~ DQB1*02:01	88	4.7
	DRB1*01:01~ DQB1*05:01	76	4.1
	DRB1*07:01~ DQB1*02:02	60	3.2
	DRB1*11:04~ DQB1*03:01	50	2.7
	DRB1*16:01~ DQB1*05:02	49	2.6
	DRB1*08:01~ DQB1*04:02	44	2.3
*A~* *C~* *B~* *DRB1~* *DQB1*	A*01:01~ C*07:01~ B*06:01~ DRB1*11:01~ DQB1*03:01	25	1.3
	A*01:01~ C*08:01~ B*07:01~ DRB1*03:01~ DQB1*04:01	24	1.3
	A*01:01~ C*08:01~ B*04:01~ DRB1*07:01~ DQB1*03:03	9	0.5
	A*02:01~ C*13:02~ B*06:02~ DRB1*07:01~ DQB1*02:02	9	0.5

## Data Availability

No new data were created or analyzed in this study. Data sharing is not applicable to this article.

## Supplementary Materials

The following supporting information can be downloaded at: https://www.mdpi.com/article/10.3390/genes16030342/s1, Table S1: Common HLA Class I and Class II Alleles in Kazakh Population of Kazakhstan.
