# Supplementary material for: Genetic Diversity and Ethnic Tapestry of Kazakhstan as Inferred from HLA Polymorphism and Population Dynamics: A Comprehensive Review
Source: Genes (Basel). 2025 Mar 15;16(3):342. doi: 10.3390/genes16030342 (PMC11941833; doi:10.3390/genes16030342)
Supplement: Supplementary file 1 [file genes-16-00342-s001.zip › genes-3514104-supplementary.pdf]

Supplementary Table S1

Common HLA Class I and Class II Alleles in Kazakh Population of Kazakhstan

Locus	Allele	Frequency	Locus	Allele	Frequency
**HLA -A**	02:01	0.1868	**HLA-B**	35:01	0.0514
	24:02	0.1529		40:02	0.0509
	01:01	0.1008		58:01	0.0491
	03:01	0.0853		15:01	0.0371
	11:01	0.0741		50:01	0.0341
	26:01	0.0498		48:01	0.0330
	31:01	0.0419		08:01	0.0327
	33:03	0.0401		35:03	0.0293
	02:06	0.0385		52:01	0.0289
	30:01	0.0339		57:01	0.0287
	68:01	0.0273		44:02	0.0286
	32:01	0.0211		18:01	0.0280
	02:07	0.0195		27:05	0.0261
	23:01	0.0193		40:01	0.0259
	24:01	0.0173		37:01	0.0218
	02:05	0.0150		44:03	0.0212
	33:01	0.0128		46:01	0.0212
	25:01	0.0123		49:01	0.0198
	29:01	0.0087		38:01	0.0180
	03:02	0.0066		54:01	0.0171
	29:02	0.0048		15:18	0.0159
	02:11	0.0034		13:01	0.0152
	30:04	0.0034		55:010	0.0132
	66:01	0.0030		14:02	0.0125
	11:02	0.0023		40:06	0.0112
	02:03	0.0023	HLA-C	06:02	0.1392
	30:02	0.0021		04:01	0.0914
	24:17	0.0018		07:02	0.0883
	02:02	0.0012		03:04	0.0755
	24:03	0.0012		01:02	0.0700
	68:02	0.0012		07:01	0.0682
	34:02	0.0011		15:02	0.0498
	69:01	0.0011		12:03	0.0482
**HLA-B**	51:01	0.0890		03:02	0.0428
	13:02	0.0623		03:03	0.0412
	07:02	0.0523		08:01	0.0385

Supplementary Table S1 (*continued*)

Locus	Allele	Frequency	Locus	Allele	Frequency
**HLA-C**	02:02	0.0310	DRB1	13:02	0.0325
	12:02	0.0277		15:02	0.0264
	07:04	0.0243		11:04	0.0214
	05:01	0.0225		10:01	0.0211
	14:02	0.0175		04:05	0.0189
	06:01	0.0145		08:03	0.0187
	08:02	0.0136		04:04	0.0173
	03:01	0.0136		08:01	0.0171
	08:03	0.0111		04:03	0.0164
	02:02	0.0310		08:02	0.0162
	12:02	0.0277		12:02	0.0157
	07:04	0.0243		14:03	0.0116
	05:01	0.0225		04:02	0.0114
	14:02	0.0175	DQB1	03:01	0.2352
	06:01	0.0145		02:01	0.1620
	08:02	0.0136		05:01	0.0912
	03:01	0.0136		03:02	0.0739
	08:03	0.0111		06:02	0.0735
DRB1	07:01	0.1299		06:01	0.0626
	03:01	0.1012		06:03	0.0514
	15:01	0.0885		03:03	0.0482
	04:01	0.0683		02:02	0.0478
	13:01	0.0616		05:02	0.0350
	1:01	0.0582		05:03	0.0280
	11:01	0.0578		04:02	0.0221
	09:01	0.0387		04:01	0.0195
	14:01	0.0382		06:04	0.0175
	12:01	0.0332		06:09	0.0128
